# The influence of parental expectations on adolescents’ physical activity: a chain mediation model of basic psychological needs and exercise motivation

**DOI:** 10.3389/fpsyg.2025.1662714

**Published:** 2025-10-09

**Authors:** Lumin Liu, Tianpei Li, Juan Long, Hwang Jin, Jae Woong Ahn

**Affiliations:** ^1^School of Physical Education, Jeonbuk National University, Jeonju, Republic of Korea; ^2^School of Economics and Management, Chongqing Electronic Information College, Chongqing, China

**Keywords:** adolescents, parental expectations, basic psychological needs, SDT, EVT, intermediate inspection

## Abstract

**Background:**

According to the World Health Organization and UNICEF, adolescents worldwide generally engage in insufficient physical activity. Parental expectations, as a social environmental factor, are considered potentially influential on adolescent physical activity, though the specific mechanisms require further clarification.

**Purpose:**

To explore the relationships among parental expectations, basic psychological needs, exercise motivation, and adolescent sports participation, aiming to provide theoretical foundations and practical guidance for youth sports engagement in China.

**Methods:**

A survey was conducted among 1,286 school-aged adolescents in eastern, central, and western China using the Parental Expectations Scale, Basic Psychological Needs Scale, Exercise Motivation Scale, and Physical Activity Scale.

**Results:**

Parental expectations significantly and positively predicted adolescent sports participation. Basic psychological needs and exercise motivation partially mediated the relationship between parental expectations and adolescent sports participation, exhibiting a chained mediating effect.

**Conclusion:**

Parental expectations exert a positive influence on adolescent sports participation. Within the family context, positive expectations can translate into external motivation for youth sports engagement. The chained mediation by basic psychological needs and exercise motivation indicates that parental expectations first satisfy adolescents’ fundamental psychological needs, thereby stimulating their exercise motivation, and ultimately promoting youth sports participation.

## 1 Introduction

Adolescents are vital to sustainable human development and form the foundation of societal progress. Reports from the World Health Organization (WHO) and UNICEF estimate that globally, approximately one in seven adolescents aged 10–19 suffers from mental health conditions, including anxiety, autism spectrum disorders, and behavioral problems, with one-third of these issues emerging before the age of 14 (United Nations, World Health Organization, and United Nations Children’s Fund, 2024). At the same time, the Report on Nutrition and Chronic Disease Status among Chinese Residents (2020), published by the State Council of China, highlights a rising trend in obesity across the population, with rates of overweight and obesity reaching 19% among children and adolescents aged 6–17, and 10.4% among those under 6 years old (State Council Information Office of the People’s Republic of China, 2020). In recent years, a substantial body of study shows that participation in physical activities not only improves adolescent mental health but also effectively promotes physical wellbeing (Laurier et al., 2024). Parental expectations were found to significantly and positively predict adolescent physical activity refers to voluntary sporting activity, typically involving engagement in exercise or sports programs, intended to improve physical fitness and support psychological and social wellbeing (Babic et al., 2014). However, despite the widely recognized benefits of adolescent Parental expectations were found to significantly and positively predict adolescent physical activity, its level in China remains suboptimal. According to findings from the 2020 Bulletin on the Status of National Fitness Activities, nearly 44.1% of young people engage in physical activity fewer than three times each week. (General Administration of Sport of China, 2022), On a global scale, roughly 81% of individuals do not achieve the advised daily target of engaging in at least 60 min of moderate to vigorous physical activity (United Nations, 2024). Therefore, uncovering the key factors influencing adolescent Parental expectations were found to significantly and positively predict adolescent physical activity, understanding the underlying mechanisms, and exploring effective strategies to increase participation is of considerable theoretical and practical importance.

Drawing upon Social Ecological Theory, adolescent development is a complex process influenced by multilevel environmental factors. As the most fundamental and enduring microsystem influencing adolescent development, the family plays a pivotal role (Steinberg, 2001). Parents not only facilitate Parental expectations were found to significantly and positively predict adolescent physical activity through explicit material support (e.g., sports equipment) and emotional support (e.g., encouragement) (Gao et al., 2024) but also shape their children’s sports values and behavioral patterns through implicit expectations. Parental expectations for athletic achievement can strengthen adolescents’ sense of competitiveness (Fredricks and Eccles, 2005), while health-oriented expectations can encourage the formation of regular Parental expectations were found to significantly and positively predict adolescent physical activity habits (Holt et al., 2008). Past research shows that what parents expect has a strong effect on how much teenagers take part in sports (Bonavolontà et al., 2021). This study looks at how parental expectations affect teenagers’ choice to join sports. It also gives real examples and ideas to help more teenagers get involved in physical activity and improve their mental and physical health.

## 2 Theoretical foundation

### 2.1 EVT

Expectancy-Value Theory (EVT) is often used to study what motivates people to take part in certain activities, like sports. It also looks at how people choose between different activities, like choosing sports or the arts. EVT says that what people achieve and what they choose to do mostly depends on two main ideas: how much they believe they can succeed and how important they think the activity is. These are called expectancy and value beliefs. (Zhu and Ma, 2017). Expectations and values interact with each other, influencing an individual’s interests and achievements. Consequently, a person’s actions are largely influenced by how likely they believe they are to succeed and by the importance they assign to the activity itself. (Parsons et al., 1984). EVT can be explained with the formula M = V × E. In this formula, M stands for motivation, which means a person’s inner drive or effort. V stands for task value, which means how important the goal is to the person. E stands for expectancy, which means how likely the person thinks they can reach the goal based on past experience (Zhao, 2009).

In this model, low task value (V) or low expectancy (E) leads to lower motivation (M). Task value comes from a person’s inner needs. Activities that meet these needs are seen as more valuable. An activity can make a need feel stronger or weaker, depending on whether the person likes it or not. Playing sports has many benefits. It can help improve health, lower anxiety and depression, and build persistence. These benefits match the needs that many teenagers have as they grow (Emmonds et al., 2024). Parents may also give messages like “sports are good for your health” or “sports matter.” These messages can help teenagers see sports as more valuable.

Expectancy reflects an individual’s assessment of their ability to succeed, typically grounded in past experiences. Sociologically, it is defined as “the individual’s perception of the likelihood that a specific behavior will lead to a particular outcome in a given context” (McClelland, 1987). As a socio-environmental influence, parental expectations can simultaneously enhance adolescents’ perceived competence (E) and value judgments (V), thereby strengthening their self-efficacy and reinforcing the perceived importance of participating in sports (Rimkute et al., 2012).

In summary, applying EVT in this study is particularly relevant, as physical activity constitutes an achievement-oriented behavior. When adolescents decide whether to engage in physical activity, they consider both their expected success (E) and the personal significance of the activity (V). Parents, as primary agents of socialization, indirectly shape both components through the expectations they communicate.

### 2.2 Self-Determination Theory (SDT)

Self-Determination Theory (SDT), developed by Ryan and Deci (2000), examines the degree to which human behavior is self-determined and volitional. SDT posits that humans are inherently proactive, with a natural inclination toward psychological growth and development, constantly seeking to overcome challenges and integrate external experiences into their self-concept (Liu and Zhang, 2010). SDT is structured around four mini-theories: Organismic Integration Theory, Basic Psychological Needs Theory, Cognitive Evaluation Theory, and Causality Orientations Theory. Central to this study is Basic Psychological Needs Theory (BPNT), a component of SDT, which posits three fundamental psychological needs: autonomy, competence, and relatedness. Autonomy means feeling free to make your own choices. Competence means feeling able and effective when dealing with the world. Relatedness means feeling close to and cared for by important people (Vansteenkiste et al., 2020). BPNT says that when these three needs are met, people become more motivated from within. This helps them stay involved in activities and show more positive behavior (Cox, 1998).

**FIGURE 1:**
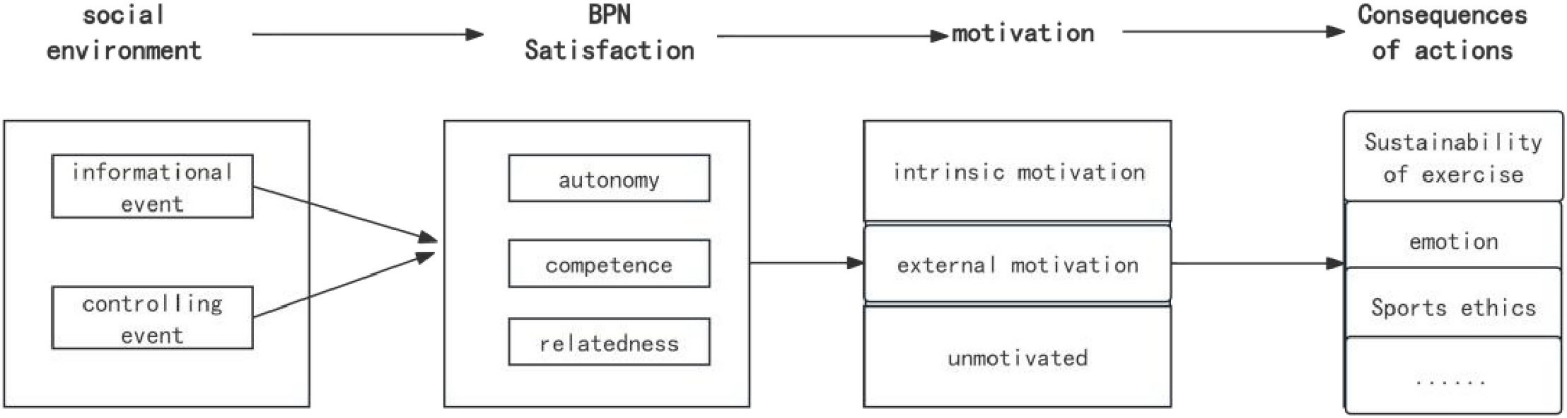
Causal path model of social environmental factors on individual behavior.

Social and environmental factors play an important role in meeting basic psychological needs. Research shows that these factors can be divided into two types: informational events and controlling events (Liu and Zhang, 2010). Informational events, such as positive feedback that affirms competence or guidance that supports skill development, satisfy basic psychological needs and bolster intrinsic motivation. In contrast, controlling events, which include rewards contingent on specific outcomes, typically undermine intrinsic motivation. The impact of parental expectations on adolescent physical activity is mediated by whether these expectations are perceived as informational or controlling. Parental expectations that convey supportive messages, such as “The impact of parental expectations on adolescent physical activity is mediated by whether these expectations are perceived as informational or controlling. can contribute to your holistic development” or “You can try this sport,” serve multiple functions. First, they affirm the adolescent’s competence, thus satisfying the need for competence. Second, they strengthen the parent-child bond through emotional resonance, addressing the need for relatedness. Third, they respect the adolescent’s freedom of choice, fulfilling the need for autonomy. Together, these elements create a synergistic effect that enhances intrinsic motivation. Thus, parental expectations, functioning as a significant socio-environmental factor, contribute to the satisfaction of adolescents’ basic psychological needs. This need satisfaction then bolsters intrinsic motivation, leading to increased frequency and persistence in physical activity (see Figure 1).

## 3 Research Hypothesis

### 3.1 The impact of parental expectations on physical activity

Parental expectations are the beliefs or aspirations that parents hold for their children’s future development, shaped by their own life experiences and knowledge (Wu, 2024). As a malleable psychological construct, parental expectations not only guide parents’ own behaviors but can also significantly influence their children’s behaviors, a phenomenon referred to as the “Pygmalion effect” (Cobos-Sanchiz et al., 2022). The mechanism of parental expectations aligns with this effect, operating through a sequence of “envisioning - expecting - acting - sensing - accepting – externalizing” (Jin, 2014). That is, the expecter (parent) forms specific expectations for the expectee (child) and anticipates corresponding behaviors. Through praise and encouragement, the expecter communicates their expectations, motivating the expectee to strive toward fulfilling them. Since parents are the expecters, children often inherently trust their judgments. Upon perceiving parental expectations, adolescents are highly likely to act accordingly. Existing research demonstrates that parental expectations significantly and positively influence adolescents’ academic achievement (Li et al., 2024) and physical activity (Lian et al., 2021).

From the perspective of Expectancy-Value Theory (EVT), parental expectations can lead adolescents to develop positive expectancy beliefs (E), such as “I am capable in sports,” and value beliefs (V), such as “Sports are beneficial for my health.” These beliefs make adolescents perceive the task as both valuable and achievable, thereby activating their exercise motivation and fostering a willingness to participate in sports. Therefore, it is plausible to hypothesize that parental expectations influence adolescent physical activity.

Furthermore, according to Self-Determination Theory (SDT), socio-environmental factors that provide informational support (e.g., positive feedback) can enhance intrinsic motivation (Leavell, 2015). For instance, when parents offer affirming messages such as “You can excel in sports’, they positively influence adolescents” intrinsic motivation. Empirical studies confirm that parental expectations significantly affect adolescent physical activity (Jaf et al., 2023). Consequently, Hypothesis H1 is proposed: Parental expectations significantly and positively influence adolescent physical activity.

### 3.2 Mediating role of basic psychological needs

According to Basic Psychological Needs Theory, individuals inherently require the satisfaction of three core psychological needs: autonomy, competence, and relatedness. Meeting these needs plays a critical role in guiding behavior and fostering mental wellbeing (Vansteenkiste et al., 2020). SDT highlights the significance of socio-environmental factors in promoting the satisfaction of these basic psychological needs. Places that help meet these needs make people feel good about themselves. They also support good health and strong relationships (Schutte and Malouff, 2021). Places that do not meet these needs can cause anxiety, depression, and other bad feelings (Liu et al., 2024). In China, parents are key participants in their children’s socialization process and play a vital role in fulfilling their children’s fundamental psychological needs. By respecting their children’s autonomy in making choices, nurturing their abilities, and enhancing their sense of belonging, parents effectively promote their healthy development while conveying their expectations (Ryan and Deci, 2017). When teenagers feel that these needs are met, they become more motivated from inside. This helps them feel better and have more positive emotions. Parental expectations are part of the social world. They play an important role in helping teenagers grow well. One study found that what parents expect is strongly linked to how well teenagers feel their basic needs are met (Liem et al., 2025).

Meeting basic psychological needs directly affects how much teenagers take part in sports (Kang et al., 2020). Gao and others say that meeting basic psychological needs helps teenagers take part in sports more and keep going longer. This happens mostly because it increases their inner motivation. When teenagers feel free to make their own choices, they see their actions as their own, not forced by others. Teenagers who feel this way about sports show more excitement and keep trying harder. In contrast, when autonomy is thwarted, adolescents may feel coerced or experience a misalignment with their values, which can lead to decreased participation or even dropout (Chen et al., 2015). Second, the satisfaction of the need for competence involves feeling effective and capable in an activity. Research conducted by Kalajas-Tilga et al. (2022) revealed a notable positive association between adolescents’ perceived level of competence and their willingness to engage in athletic activities. Third, the satisfaction of the need for relatedness entails forming positive connections and feeling understood and supported. A sense of belonging within the sports context is positively associated with participation behavior. Specifically, when adolescents feel accepted and form meaningful social connections through sports, their engagement in sports activities significantly increases (White et al., 2018). Thus, the satisfaction of these basic psychological needs fosters greater motivation and wellbeing, leading to stronger intentions for physical activity. Accordingly, Hypothesis H2 is proposed: Basic psychological needs mediate the positive effect of parental expectations on adolescent Campaign participation.

### 3.3 Mediating role of exercise motivation

Motivation is the psychological force that arouses, sustains, and directs behavior toward a goal (Atkinson, 1964). EVT posits that an individual’s expectations of success and the perceived value of a task are primary determinants of their motivation and subsequent behavior. Parental expectations, as significant socializing influences, are instrumental in influencing the development of these cognitive perceptions among adolescents. The mechanism through which parental expectations influence adolescent physical activity operates primarily through two pathways: First, by shaping expectancy beliefs. Parents’ beliefs in their adolescent’s abilities and the expectations they convey directly affect the adolescent’s perception of their likelihood of success in sports. Positive and attainable parental expectations can enhance adolescents’ self-efficacy, thereby stimulating their exercise motivation (Hua et al., 2024). Second, by shaping the perceived value of the task. The attitudes parents hold toward physical activity significantly influence how adolescents value such activities. When parents highlight the intrinsic enjoyment and personal development aspects of sports (e.g., “Sports help you grow healthily”), they foster greater intrinsic motivation and interest in their children (Gao et al., 2024). Thus, it is reasonable to hypothesize that parental expectations significantly influence adolescents’ exercise motivation.

**FIGURE 2:**
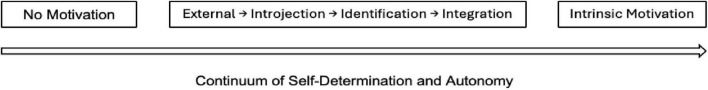
Continuum of motivational change.

Exercise motivation is defined as the psychological impetus that drives individuals to initiate and sustain physical activity, serving as the immediate precursor to physical activity behavior (Liu et al., 2011). According to Self-Determination Theory (SDT), motivation exists along a continuum of relative autonomy, spanning from amotivation, through different types of extrinsic motivation, to intrinsic motivation (Liu et al., 2025) (see Figure 2). Different types of motivation have distinct effects on the initiation and persistence of physical activity. Individuals who are amotivated lack the intention to engage in physical activity and may view it as valueless, resulting in non-participation (Ryan and Deci, 2017). Those motivated extrinsically engage in sports to obtain rewards or avoid negative consequences; although this can sustain participation temporarily, it typically diminishes once the external incentives are withdrawn (Abós et al., 2021). Intrinsically motivated individuals engage in sports voluntarily and with a sense of autonomy. Empirical studies indicate that they demonstrate superior self-regulation and sustain higher levels of persistence, even in the face of challenges (Vasconcellos et al., 2020).

To summarize, parental expectations are likely to influence adolescent physical activity through their impact on exercise motivation, which in turn promotes greater engagement. Accordingly, Hypothesis H3 proposes that the link between parental expectations and adolescents’ involvement in sports is positively influenced through the mediating role of exercise motivation.

### 3.4 Serial mediation model

According to SDT, the extent to which basic psychological needs are satisfied plays a crucial role in shaping the type of motivation individuals develop. When adolescents’ basic psychological needs are met, they are more likely to cultivate intrinsic motivation. Specifically, these needs influence exercise motivation through three primary pathways. First, autonomy: when individuals view their physical activity as self-directed rather than coerced, they are more inclined to develop intrinsic motivation [citation (Teixeira et al., 2012)]. Second, competence: achieving skill enhancement or meeting goals in sports fulfills the need for competence, thereby promoting intrinsic motivation [citation (Vlachopoulos and Michailidou, 2006)]. Third, relatedness: physical activity often takes place in social contexts, and positive interactions with teammates satisfy the need for relatedness, making the experience pleasurable and meaningful [citation (Chen et al., 2015)]. Consequently, the satisfaction of basic psychological needs significantly impacts exercise motivation.

A comprehensive review of the literature highlights established associations between parental expectations and the satisfaction of basic psychological needs, between need satisfaction and exercise motivation, and between exercise motivation and actual physical activity. However, there is a notable gap in research that thoroughly investigates the intricate interrelationships among these four variables concurrently. To address this, the current study, primarily informed by SDT and augmented by Expectancy-Value Theory (EVT), adopts a theoretical model that posits a sequential relationship from socio-environmental factors to basic psychological needs, motivation, and ultimately behavioral outcomes, as outlined in Figure 1. In this context, parental expectations, as a pivotal socio-environmental determinant, affect the degree to which adolescents’ basic psychological needs are satisfied. The fulfillment of these needs, in turn, enhances intrinsic motivation, which leads to more self-determined and enduring participation in sports.

Thus, we formulate Hypothesis H4: Parental expectations influence adolescents’ engagement in sports through the serial mediation of basic psychological needs satisfaction and exercise motivation.

## 4 Research methods

### 4.1 Participants

This study employed convenience sampling to select in-school adolescents from eastern, central, and western China as research subjects. Six secondary schools were randomly selected from each region’s rural areas, urban-rural fringe zones, and urban centers (including three general secondary schools and three sports-specialized secondary schools), totaling 18 schools. Each school included a portion of ethnic minority students as research subjects. Inclusion criteria were: (1) Aged 12–18 years; (2) Continuous participation in organized sports activities (e.g., school team training, after-school sports clubs) for at least six months; (3) No severe physical or mental illnesses. The final valid sample comprised 1,286 participants (637 males, 649 females), with a mean age of 14.82 ± 1.76 years. Of the participants, 60.4% (*n* = 777) were junior high school students, and 39.6% (*n* = 509) were senior high school students. The recruitment process was coordinated through school administrations and physical education departments. Questionnaires were administered by class after obtaining informed consent from parents. Participation was anonymous and took approximately 15–20 min. All procedures adhered to the ethical principles of the Declaration of Helsinki. The study protocol received approval from the Jeonbuk National University Institutional Review Board (Approval No. KIRD-2024-03-31-J-E-346).

### 4.2 Measures

#### 4.2.1 Parental expectations scale

The Parental Expectations Questionnaire, developed by Cheng (2010), was used to measure parental expectations. The scale includes five dimensions—Academic Performance, Future Achievement, Behavioral Standards, Social Adaptation, and physical activity—totaling 24 items, with six being reverse-scored. Responses were recorded on a five-point Likert scale (1 = “Strongly Disagree” to 5 = “Strongly Agree”). Higher total scores indicate higher levels of parental expectations. In this study, the scale was administered to the parents of the adolescents. The overall Cronbach’s α coefficient was 0.943. Confirmatory Factor Analysis (CFA) indicated good structural validity (χ^2^/df = 1.708, CFI = 0.986, TLI = 0.984, RMSEA = 0.023, SRMR = 0.038), supporting the scale’s reliability for this sample.

#### 4.2.2 Basic Psychological Needs Scale For adolescents

The Basic Psychological Needs Scale for adolescents, specifically the Chinese version revised by Liu et al. (2013), was used to measure adolescents’ basic psychological needs. This scale includes three dimensions: Autonomy, Competence, and Relatedness. Sample items include “I can decide how to complete tasks myself” for Autonomy, “I feel capable of doing well” for Competence, and “I feel respected by my peers” for Relatedness. Participants responded using a five-point Likert scale, ranging from 1 (Strongly Disagree) to 5 (Strongly Agree). The scale demonstrated high internal consistency in this study, with a Cronbach’s α coefficient of 0.925. Confirmatory Factor Analysis (CFA) was conducted to assess the scale’s structural validity, revealing a good model fit with the following indices: χ^2^/df = 1.474, CFI = 0.993, TLI = 0.992, RMSEA = 0.019, and SRMR = 0.034.

#### 4.2.3 Simplified exercise motivation scale

The Simplified Exercise Motivation Scale, specifically the 15-item version revised by Chen et al. (2013), was used to assess exercise motivation. This scale encompasses five dimensions: Enjoyment Motivation, Competence Motivation, Appearance Motivation, Health Motivation, and Social Motivation. Sample items include “Exercise makes me happy” for Enjoyment Motivation, “I want to improve my sports skills” for Competence Motivation, “I want to improve my physique” for Appearance Motivation, “Exercise keeps me healthy” for Health Motivation, and “I can spend time with friends” for Social Motivation. Each dimension consists of three items, and participants rated their responses on a five-point Likert scale ranging from 1 (Strongly Disagree) to 5 (Strongly Agree). The scale demonstrated high internal consistency in this study, with a Cronbach’s α coefficient of 0.919. Confirmatory Factor Analysis (CFA) was conducted to assess the scale’s structural validity, revealing a good model fit with the following indices: χ^2^/df = 1.520, CFI = 0.994, TLI = 0.993, RMSEA = 0.020, and SRMR = 0.034.

#### 4.2.4 Physical Activity Rating Scale

The Chinese version of the Physical Activity Rating Scale (PARS-3), updated by Liang (1994), was used to measure how much teenagers take part in sports. This scale is used in many countries to check exercise habits. It has shown to be reliable and accurate in past studies. The scale has three parts: Intensity, Frequency, and Duration. Intensity means how hard the exercise is, like breathing and sweating. It is rated from 1 (Light) to 5 (Very Hard or Long). Frequency means how many times a week a person exercises, rated from 1 to 5. Duration means how long each exercise session lasts in minutes. It is also rated from 1 to 5 using set time ranges. The total physical activity score was calculated using the formula: Intensity × Frequency × (Duration-1). Higher total scores indicate greater levels of physical activity. The scale has been successfully applied in Chinese student populations, further supporting its validity for this study. In this study, the scale demonstrated acceptable internal consistency, with a Cronbach’s α coefficient of 0.831.

### 4.3 Data analysis

Data analyses, including descriptive statistics, correlation analysis, and testing for common method bias (CMB), were conducted using SPSS 26.0. Additionally, the serial mediation model was tested using the SPSS macro PROCESS (Model 6, Hayes).

### 4.4 Normality test

According to the normal distribution test method proposed by WEST (Kim, 2013), a variable is considered to follow a normal distribution when its skewness falls between −2 and +2 and its kurtosis falls between −7 and +7. In this study, the skewness of physical activity exceeded 1. However, with a large sample size, minor deviations in skewness or kurtosis do not significantly impact the normality test. Therefore, the variables in this study can be considered normally distributed, satisfying the prerequisite for data analysis (Table 1).

**TABLE 1:** Normality test.

Variable	Skewness	Kernel
Parental expectations	−0.094	−1.200
Physical activity	1.214	0.245
BPN satisfaction	−0.018	−0.046
Exercise motivation	0.001	−0.225

## 5 Results

### 5.1 Common method bias test

Harman’s single-factor test was conducted on all 60 items. The unrotated factor solution extracted four factors. The first factor explained 17.274% of the total variance, well below the critical threshold of 40% (Tang and Wen, 2020). This suggests that common method bias is unlikely to have significantly influenced the results.

### 5.2 Correlation analysis

Table 2 presents descriptive statistics and bivariate correlations among the main study variables. Parental Expectations (M = 2.917, SD = 1.009), physical activity (M = 28.093, SD = 32.293), basic psychological need satisfaction (M = 2.838, SD = 1.049), and Exercise Motivation (M = 2.857, SD = 1.105) were significantly and positively intercorrelated. Parental expectations correlated positively with physical activity (r = 0.208, *p* < 0.01), basic need satisfaction (r = 0.191, *p* < 0.01), and exercise motivation (r = 0.207, *p* < 0.01). Basic need satisfaction correlated positively with physical activity (r = 0.216, *p* < 0.01) and exercise motivation (r = 0.269, *p* < 0.01). Exercise motivation also correlated positively with physical activity(r = 0.193, *p* < 0.01). These results provide preliminary support for subsequent serial mediation analysis.

**TABLE 2:** Descriptive statistics and correlations among key variables.

Variable	M	SD	Parental exp.	Sports part.	BPN sat.	Exercise mot.
Parental expectations	2.917	1.009	1	1	1	1
Physical activity	28.093	32.293	0.208** (0.155∼0.260)			
BPN satisfaction	2.838	1.049	0.191** (0.138∼0.243)	0.216** (0.163∼0.267)		
Exercise motivation	2.857	1.105	0.207** (0.154∼0.258)	0.193** (0.140∼0.245)	0.269** (0.218∼0.319)	

**P < 0.01. BPN Sat., Basic Psychological Need Satisfaction. The numbers in parentheses represent confidence intervals.

### 5.3 The impact of parental expectations on adolescent physical activity

Before looking at the direct effect of parental expectations on adolescent physical activity, we need to test measurement invariance on the scales. This study used samples from eastern, central, and western China, and there may be cultural differences between these regions. These differences could lead to different understandings of the measurement tools. So, it is important to test measurement invariance to make sure cross-group comparisons are valid and reliable. If measurement invariance is not met, the differences we see may only come from bias in the tools, not from real psychological differences. Therefore, this study sequentially examined configuration invariance, scale loading invariance, and structural covariance invariance, and determined the validity of invariance based on commonly used fit indices such as RMSEA < 0.05.

As shown in Table 3, for the chi-square test, the fit indices of the invariance model were χ^2^ = 1665.258, df = 832, *p* < 0.001, χ^2^/df = 2.002. The scale loading model gave results that were the same as the invariance model. The structural covariance model gave χ^2^ = 1740.198, df = 842, *p* < 0.001, χ^2^/df = 2.067. The chi-square value was significant, but because it is sensitive to sample size, other fit indices were checked. For absolute fit indices, RMR values were 0.155, 0.155, and 0.153. GFI values were close to 0.91 (GFI = 0.911 for the configuration-invariance and scale loading models; GFI = 0.907 for the structural covariance model), showing a good fit. For comparative fit indices, CFI and IFI were 0.938 in both the configuration-invariant and scale-loading models, and 0.933 in the structural covariance model. These values were close to the suggested cutoff of 0.95, showing excellent fit. TLI was 0.938 (configuration invariance and Metric Invariance models) and 0.934 (structural covariance model), both higher than the minimum standard of 0.90. NFI values were between 0.877 and 0.883, which was a little below the ideal standard of 0.90 but still within an acceptable range.

**TABLE 3:** Measurement invariance test.

Model	CMIN	DF	RMR	CMIN/DF	GFI	RMSEA	CFI	NFI	IFI	TLI	ΔCFI	ΔRMSEA
Configuration-invariant model	1665.258	832	0.155	2.002	0.911	0.028	0.938	0.883	0.938	0.938		
Scale loading model	1665.258	832	0.155	2.002	0.911	0.028	0.938	0.883	0.938	0.938	0.000	0.000
Structural covariance model	1740.198	842	0.153	2.067	0.907	0.029	0.933	0.877	0.933	0.934	−0.005	0.001

In summary, although the chi-square value is significant, multiple fit indices indicate that the configuration-invariant model and the scale loading model exhibit comparable fit and overall outperform the independent model. The structural covariance model also demonstrates acceptable fit. Therefore, the scales used in this study maintain measurement invariance across different regions and are suitable for cross-regional comparative analysis.

Using the mediation testing method by Wen et al. (2004), regression analysis was done with parental expectations as the independent variable and adolescent physical activity as the dependent variable. The results showed that parental expectations had a clear positive effect on adolescent physical activity (β = 0.208, t = 7.634, *P* = 0.000). This study looked at the effects of parental expectations on different parts of physical activity. So, we tested if parental expectations could separately affect exercise intensity, exercise frequency, and exercise duration. Gender, age, and grade level were controlled, and regression analysis was run with exercise intensity, frequency, and duration as dependent variables (Table 4). The results showed that parental expectations had the strongest effect on exercise frequency, then on exercise intensity, and the weakest effect on exercise duration. The variance inflation factor (VIF) values in the model were from 1 to 1.003, which showed there was no multicollinearity among the variables. The detailed results are as follows:

(1)    When exercise intensity was the dependent variable, parental expectations exerted a significant positive influence on exercise intensity in adolescents’ physical activity (β = 0.202, *P* < 0.001).
(2)    When exercise frequency was the dependent variable, parental expectations exerted a significant positive influence on exercise intensity in adolescents’ physical activity (β = 0.238, *P* < 0.001).
(3)    When exercise duration was the dependent variable, parental expectations exerted a significant positive influence on exercise intensity in adolescents’ physical activity (β = 0.184, *P* < 0.001).

**TABLE 4:** Direct effects test.

Variable	Exercise intensity	Exercise frequency	Exercise duration
Gender	−0.035	−0.013	−0.022
Age	−0.021	−0.030	−0.031
Grade level	0.039	0.069	0.076
Parental expectations	0.202***	0.238***	0.184***
R^2^	0.043	0.061	0.040
ΔR^2^	0.040	0.058	0.037
F	14.466***	20.925***	13.270***

***P < 0.001.

### 5.4 Serial mediation model test

Using PROCESS Model 6 (controlling for gender, age, and grade), the serial mediation involving basic psychological need satisfaction and exercise motivation in the relationship between parental expectations and physical activity was tested. Results are presented in Tables 5, 6.

**TABLE 5:** Regression results for the serial mediation model.

Predictor	Physical activity		BPN satisfaction		Exercise motivation		Physical activity	
	β	t	β	t	β	t	β	t
Parental expectations	0.206	7.581***	0.190	6.914***	0.156	5.877***	0.154	5.611***
BPN Satisfaction					0.236	8.735***	0.152	5.471***
Exercise motivation							0.114	4.066***
Gender	−0.071	−1.302	0.035	0.635	0.048	0.902	−0.082	−1.55
Age	−0.01	−0.744	−0.005	−0.327	0.013	1.001	−0.011	−0.813
Grade	0.075	4.025***	0.025	1.315	0.048	2.668*	0.065	3.557**
R	0.238		0.196		0.322		0.315	
R^2^	0.057		0.038		0.104		0.099	
F	19.264***		12.750***		29.704***		23.498***	

BPN Sat., Basic Psychological Need Satisfaction.

*p < 0.05,

**p < 0.01,

***p < 0.001.

**TABLE 6:** Bootstrap test of indirect effects.

Path	Efficiency value	BootSE	BootLLCI	BootULCI
Total effect (parental exp.→physical activity)	0.209	0.027	0.153	0.259
Direct effect (parental exp.→physical activity)	0.154	0.027	0.100	0.208
Total indirect effect	0.052	0.011	0.032	0.073
Parental exp.→BPN Sat.→physical activity	0.029	0.008	0.015	0.046
Parental exp.→exercise mot.→physical activity	0.018	0.006	0.007	0.032
Parental exp.→BPN Sat.→exercise mot.→physical activity (serial med.)	0.005	0.002	0.002	0.009

BPN Sat., Basic Psychological Need Satisfaction. Boot SE, bootstrap standard error; LLCI/ULCI, lower/upper level of 95% bias-corrected confidence interval (5,000 bootstrap samples). Confidence intervals excluding zero indicate significance.

Hierarchical regression analysis (Table 5) showed that after controlling for gender, age, and grade, parental expectations had a significant direct effect on physical activity (β = 0.206, t = 7.581, *p* < 0.001), explaining 5.7% of the variance (F = 19.264, *p* < 0.001). The mediation path analysis revealed: (1) Parental expectations significantly and positively predicted basic need satisfaction (β = 0.190, t = 6.914, *p* < 0.001); (2) Basic need satisfaction had a significant direct effect on physical activity (β = 0.236, t = 8.735, *p* < 0.001) and also indirectly influenced participation by enhancing exercise motivation (β = 0.152, t = 5.471, *p* < 0.001); (3) Exercise motivation independently predicted sports participation (β = 0.114, t = 4.066, *p* < 0.001). When all three predictors were included simultaneously, the model’s explanatory power increased to 9.9% (F = 23.498, *p* < 0.001). The direct effect of parental expectations remained significant (β = 0.154, t = 5.611, *p* < 0.001), indicating partial mediation by basic need satisfaction and exercise motivation. Grade level showed significant effects across several models (β = 0.048–0.075, *p* < 0.05), suggesting the need to control for educational stage in future research.

**FIGURE 3:**
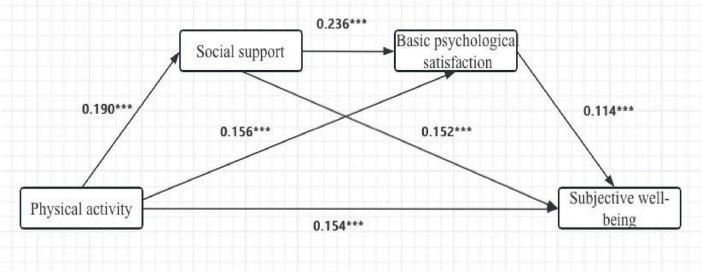
Chain-based intermediary model diagram. ***p < 0.001.

Bootstrap analysis (Table 6) confirmed the total effect of parental expectations on physical activity [Effect = 0.209, Boot SE = 0.027, 95% CI (0.153, 0.259)] and its significant direct effect [Effect = 0.154, Boot SE = 0.027, 95% CI (0.100, 0.208)]. Three significant indirect pathways were identified: (1) Through basic need satisfaction alone (Effect = 0.029, Boot SE = 0.008, 95% CI (0.015, 0.046)]; (2) Through exercise motivation alone [Effect = 0.018, Boot SE = 0.006, 95% CI (0.007, 0.032)]; (3) Through the serial path Parental Exp. → BPN Sat. → Exercise Mot. → Sports Part. [Effect = 0.005, Boot SE = 0.002, 95% CI (0.002, 0.009)]. The 95% confidence intervals for all three paths excluded zero, confirming their statistical significance. The total indirect effect was 0.052 [Boot SE = 0.011, 95% CI (0.032, 0.073)], accounting for 24.88% of the total effect (Figure 3). This underscores the important mediating roles of basic need satisfaction and exercise motivation, with the mediation via basic needs being the most substantial. These findings provide empirical support for understanding the intrinsic mechanisms linking parental expectations to adolescent physical activity.

## 6 Discussion

### 6.1 Direct effect of parental expectations on adolescent physical activity

The results demonstrate that parental expectations significantly and positively predict adolescent Physical activity, aligning with findings by Fredricks and Eccles (2005). This study innovatively employs EVT theory as one of its analytical frameworks, positing that parental expectations serve as a key external source shaping adolescents’ task value and success expectations in physical activity. When parents hold high expectations, they actively communicate the importance of physical activity and express confidence in their child’s abilities, thereby reinforcing the value and feasibility of physical activity. This transmission of values may lead adolescents to perceive physical activity as a highly valuable endeavor, fostering their belief in their capability to engage successfully and derive benefits or enjoyment, thereby enhancing their initiative. Notably, the direct effect of parental expectations on Physical activity remained significant even after accounting for basic need satisfaction and exercise motivation. This suggests that beyond the indirect pathways involving basic need satisfaction and exercise motivation, factors inherent to parental expectations—such as goal setting and resource provision—exert an independent and substantial influence on adolescent sports behavior. This effect may be particularly potent within the Chinese cultural context, where traditional aspirations like “hoping one’s son becomes a dragon” or “hoping one’s daughter becomes a phoenix” imbue parental expectations with significant authority and influence (Leung and Shek, 2011). This study did not look at the effect of parental demographic factors on children. But other scholars have found that parental expectations for children’s physical activity are shaped by things like socioeconomic status, income, and education level. Cheng (2010) research showed that parents who worked as administrators or teachers had the highest expectations for their children. This may be because they had higher education and regular work schedules, which let them give both money and time to their children’s sports activities. Parents with higher incomes often focus on both learning and physical growth, supporting balanced development. In contrast, parents with lower incomes may not have the same resources, which can limit how well they can carry out their educational goals for their children. While the national “Healthy China Initiative” and the societal re-evaluation of sports’ value have increased parental emphasis on children’s physical development, the potential negative consequences of unrealistic or coercive expectations—such as excessive pressure, anxiety, or reactance—warrant careful consideration and further investigation through longitudinal designs.

### 6.2 Mediating role of basic psychological needs

The findings confirm that the fulfillment of basic psychological needs partially mediates the connection between parental expectations and engagement in sports. According to SDT, the lack of satisfaction of inherent needs—namely autonomy, competence, and relatedness—is central to the development of intrinsic motivation. (Leo et al., 2022). In the context of adolescent sports, parental expectations serve as a socio-environmental influence, not by directly dictating behavior, but by shaping an environment that either facilitates or hinders the satisfaction of these basic psychological needs, thereby indirectly influencing motivation and participation. This result agrees with research by Ruiz et al. (2021), Trigueros et al. (2019). They show that social and environmental factors affect motivation (Ruiz et al., 2021; Trigueros et al., 2019). Competence: How parents talk about their expectations affects how teens feel about their abilities. When parents use kind and supportive words, teens see their value and potential in sports. This helps them believe more in their skills. Parental recognition and praise for sports involvement further reinforce this perception of competence. Relatedness: The expression of parental expectations is closely associated with adolescents’ need for relatedness, defined as the desire for stable, positive emotional bonds and feelings of care and acceptance (Allen et al., 2022). When parents offer emotional understanding and support, enabling adolescents to feel accompanied and nurtured by their family within the sports context, their relatedness needs are fulfilled. This increases the enjoyment of sports and positions participation as an element of positive parent-child interaction, thereby enhancing engagement (e.g., watching games or exercising together). Autonomy: The influence of parental expectations on autonomy is particularly critical. Although inherently. directive, expectations communicated in a manner that respects adolescent” choices and interests, thereby promoting self-determination, can effectively support or even enhance their autonomy. When adolescents perceive physical activity as self-initiated rather than imposed by parents, their intrinsic motivation is strengthened, resulting in more persistent and frequent engagement.

### 6.3 Mediating role of exercise motivation

The results indicate that exercise motivation plays a partially mediating role in the association between parental expectations and adolescents’ engagement in sports activities. According to Organismic Integration Theory (OIT), a sub-theory of Self-Determination Theory (SDT), motivation exists on a continuum from amotivation to intrinsic motivation, characterized by varying degrees of autonomy (Ryan and Deci, 2024). Parents who emphasize the multifaceted benefits of sports—such as enhancing physical and mental health and fostering resilience (Mahindru et al., 2023)—significantly increase their children’s perceived value of physical activity. SDT posits that when individuals internalize external influences, such as parental expectations, and integrate them with their sense of self, extrinsic motivation can evolve into intrinsic motivation (Ryan and Deci, 2002). Research by Mota and Queirós (1996) highlights parents as the primary agents of sports socialization, with their attitudes significantly influencing their children’s motivation. Parents help teenagers learn the value of sports by showing good examples, giving advice, and explaining why sports matter. This changes teens’ motivation from “I have to” to “I want to,” which builds strong inner motivation. The study also uses Expectancy-Value Theory (EVT). It says that choosing to do something and sticking with it depends on how much people expect to succeed and how much they value the task. When parents trust their child’s sports skills and have positive expectations, teens are more likely to believe in themselves. This belief, “I can do it,” makes them more confident to face sports challenges. It also encourages them to try harder and stay involved. In conclusion, the influence of parental expectations on adolescent physical activity is mediated through complex motivational processes, involving both the internalization of values and the enhancement of self-efficacy, rather than through direct or singular pathways.

### 6.4 Serial mediating role of basic psychological needs and exercise motivation

This study identified a significant serial mediation pathway: Parental Expectations → Basic Psychological Need Satisfaction → Exercise Motivation → Physical activity. Parental expectations, as a key socio-environmental factor, exert their influence not directly but through a sequence of internal psychological processes. From an SDT perspective, basic psychological needs serve as the crucial link connecting socio-environmental factors to individual motivation. This study demonstrates that parental expectations, by satisfying adolescents’ needs for autonomy, competence, and relatedness, subsequently promote the development of adaptive exercise motivation, aligning with findings by Teixeira et al. (2012). Parental expectations can have both good and bad effects, so they need careful study. The data from this study show that parental expectations have some positive effects. But other research shows that too many or unrealistic expectations can hurt children’s growth (Chen et al., 2022; Sakaki et al., 2024). They can also become sources of stress and lower adolescents’ motivation. This pressure may come from parents not respecting their children’s independence or from setting expectations that are too high. In these cases, adolescents join sports because of outside pressure, and the activity loses its natural fun and value (Sakaki et al., 2024). Specifically, appropriate parental expectations foster a supportive psychological climate, enabling adolescents to experience autonomy, competence, and connection during physical activity, thereby enhancing need satisfaction. Satisfied needs, in turn, catalyze stronger and more autonomous forms of motivation (Fraser-Thomas et al., 2008; Pelikan et al., 2021). Exercise motivation then directly influences the frequency, intensity, and persistence of physical activity. The significant positive effect of autonomous motivation on physical activity (β = 0.114, *p* < 0.001) underscores that when adolescents are driven by intrinsic interest or personal value, they exhibit greater initiative and persistence in their physical activity. The significant serial mediation model “Parental Expectations → Basic Psychological Need Satisfaction → Exercise Motivation → Physical activity” clearly delineates the sequential pathway through which parental expectations influence physical activity. Specifically, parental expectations first shape the adolescents’ experiences of need satisfaction within the sports context. These experiences, in turn, influence the quality of their exercise motivation. Finally, the degree of autonomous motivation predicts the level of physical activity.

In summary, this study validated a chain-mediated model linking basic psychological needs fulfillment and exercise motivation based on EVT and SDT theories. This not only elucidates the intrinsic mechanism through which parental expectations influence physical activity but also indirectly verifies the applicability of EVT and SDT theories.

## 7 Research limitations and future directions

To begin with, this research primarily employed a cross-sectional survey approach to gather and examine data at specific moments in time. While such a design is useful for identifying associations among variables, it presents limitations in establishing definitive causal links. To address this issue, future investigations might adopt a longitudinal design, allowing variables to be assessed repeatedly across multiple time points, thereby providing stronger evidence for causal inference.

Secondly, the study did not look at parental expectations in relation to different parts of their children’s lives, so parental expectations cannot be seen as always having a positive effect. More research is needed, for example, to see if very high expectations about school performance or future success may reduce adolescents’ physical activity. The participants in this study were from China, and the sample may have been limited by cultural factors in the region. When applying the results to other countries or regions with different cultures, the general use of the findings needs more thought.

Lastly, the present research was conducted within the framework of Chinese cultural norms. In this context, parental expectations are widely regarded as influential in shaping adolescent development. However, limited attention has been given to how specific cultural features—such as collectivist values and elevated parental aspirations—affect the way these expectations are communicated and how young people internalize or respond to them. To gain deeper insight, future studies may benefit from incorporating qualitative methodologies to explore how culturally rooted expectations influence adolescents’ participation in sport, thereby contributing to more culturally nuanced theoretical models.

## 8 Conclusion and implications

### 8.1 Conclusion

#### 8.1.1 Research conclusion

(1)    Parental expectations have a strong positive effect on teenagers taking part in sports. This means that in family education, parents’ good hopes for their children’s sports growth can become outside motivation to get them involved. Later, this can turn into the children’s own sense of value.
(2)    Basic psychological needs and exercise motivation each partly explain how parental expectations affect teenagers’ physical activity. When basic needs are met, teens’ motivation for exercise grows inside them, which helps them join sports more actively.
(3)    This study showed a step-by-step effect: parental expectations first meet teenagers’ basic needs, then boost their motivation to exercise, and finally lead them to take part in sports. This finding combines ideas from SDT and EVT. It also shows how family factors work inside the mind to affect teens’ sports behavior. This helps explain better how expectations change into actions.
(4)    This study used a questionnaire survey with 1,286 adolescents from eastern, central, and western China. The large sample makes the results more representative and helps fix the limits of studies that use only small or local groups.

### 8.2 Implications

#### 8.2.1 Research implications

(1)    This study adds to the use of Self-Determination Theory (SDT) and Expectancy-Value Theory (EVT) in studying how teenagers join sports. It combines key ideas from both theories. It shows that parents’ expectations, as part of the social environment, directly affect teenagers taking part in sports. More important, it explains the path from expectations to needs, then motivation, and finally behavior. This helps us understand how motivation to join sports is formed.
(2)    This study tested and confirmed that basic psychological needs and exercise motivation work step by step between parental expectations and teenagers’ physical activity. This gives a clearer view of how motivation forms in sports. It also offers a model for future studies about how other social factors affect sports behavior.
(3)    Parents should share their expectations in a fair way. They should not put too much pressure or make unrealistic demands. Instead, they should use more support and encouragement to build a good family sports environment. Parents should use a more independent parenting style. They should give needed guidance but also let adolescents make their own decisions. This way, adolescents’ need for independence is met and their inner motivation is kept. This paper mainly looks at the good effects of parental expectations. But these expectations may follow an inverted U-shape. When expectations go beyond the best level and become too high, they can cause stress and anxiety.
(4)    This study shows that large-scale psychological and behavioral surveys with adolescents in China are possible and useful. Future research can use this sample to do follow-up studies over time.

## Data Availability

The raw data supporting the conclusions of this article will be made available by the authors, without undue reservation.
